# Using system dynamics mapping to explore synergy in an equity-focused obesity prevention framework

**DOI:** 10.3389/fpubh.2025.1525224

**Published:** 2025-02-20

**Authors:** Irene E. Headen, Tiffany M. Eaton, Shiriki K. Kumanyika

**Affiliations:** ^1^Department of Community Health and Prevention, Drexel University Dornsife School of Public Health, Philadelphia, PA, United States; ^2^Council on Black Health Inc., Raleigh, NC, United States

**Keywords:** health equity, system dynamics, frameworks, obesity prevention, childhood obesity, systems change, policy change, environmental change

## Abstract

**Introduction:**

Addressing health inequities across chronic diseases is a critical public health objective, and policy, systems, and environmental (PSE) change approaches are integral to achieving this goal. However, assumptions about mechanisms of effect or population salience of PSE approaches do not necessarily generalize to inequitable social and economic contexts, partially due to limited ability to operationalize the dynamic complexity of such contexts. Systems thinking applications have the potential to characterize this complexity and improve understanding of where and how to intervene.

**Methods:**

The Getting to Equity in Obesity Prevention Framework (GTE) posits a theory of change involving PSE-related considerations for achieving equity grouped into four categories with a general systems feedback structure. We used systems mapping with a case study to explore the anticipated synergy across categories of the GTE. Data were extracted from a narrative account of childhood obesity prevention initiatives in a predominantly African American and Hispanic, urban public-school district: the Philadelphia Childhood Obesity Declines Project. Project documentation described PSE strategies and contextual influences thought to have contributed to concurrently observed declines in child obesity prevalence and disparities in this population.

**Results:**

Our final dynamic framework, which was anchored by Philadelphia's Universal Feeding Pilot for school meals, identified synergies among intervention strategies. The systems map revealed how planned and unplanned processes accumulated to align with the observed disparities reductions in the participating school district, consistent with the GTE theory of change. Community context dynamics, which evolved over time, were prominent features of the map.

**Discussion:**

This case study enhances the utility of the GTE framework when paired with systems mapping enabled by detailed documentation of PSE initiatives and relevant contextual influences. This suggests that prospective mapping of considerations prompted by the GTE could improve anticipation of unplanned pathways, intervention design, and implementation and supports a need for greater priority for using systems mapping or other systems science tools and methodologies in obesity-prevention research and practice.

## 1 Introduction

Policy, systems, and environmental change (PSE) approaches are recognized as critical to addressing health disparities in the context of social disadvantage, limited economic resources, and constrained community power in economic and policy spheres (1, 2). Ideally, such approaches would address societal inequities in ways that both lower overall prevalence of the chronic disease disparities that they target, such as in obesity, and reduce disparities as well. In the case of obesity, however, for a variety of reasons, efforts to change what have been termed “obesigenic” environments may not achieve the intended level of effectiveness in the social, economic, cultural, or policy contexts that affect priority populations^1^ for disparities reduction (3), inadvertently perpetuating disparities or allowing prevalence gaps to increase (4–7). The recognition of this challenge has grown with increasing documentation of the role of structural and societal factors, past and current, in generating and perpetuating social and economic inequities in health opportunities (8, 9). Assumptions about pathways to change or the salience of existing approaches in the priority population context may not apply because of the differences in the availability and mix of resources and opportunities in marginalized communities that result from these persistent social and economic inequities (10). Challenges affecting disinvested communities may result in inadequate reach of the approach, feasibility issues with implementation, or the potential for unfavorable side effects. Additionally, the complexity of generating valid evidence about what works to advance obesity prevention in priority populations may discourage much needed research and research funding (11).

Longstanding concerns in this respect have been amplified by evidence of persistent increases in prevalence and widening of obesity disparities when comparing people classified as non-Hispanic Black or Mexican American^2^ to those classified as non-Hispanic white, including both adults and youth in some age or age-sex subgroups (12, 13). Moreover, with respect to the effectiveness of PSE interventions, data from the longitudinal Healthy Communities Study of more than 5,000 children in 130 U.S. communities suggested widening gaps in body mass index (BMI) trajectories despite potential exposure to concurrent PSE interventions (14). BMI trends were less favorable in communities with substantial proportions of African American or Hispanic children or families with lower incomes and in the Southeastern U.S. compared with trends for children in predominantly White, higher-income communities in the Northeast (14, 15). This suggested greater uptake, effectiveness, or both in the latter communities. Relevant PSE interventions must, by definition, focus on drivers within complex dynamic systems that influence obesity inequities through eating and physical activity opportunities and behaviors, taking both favorable and unfavorable influences into account (16). Achieving the desired reduction of obesity inequities will remain elusive without being able to interrogate the complex dynamic systems that characterize the contexts of these community differences.

**Figure 1:**
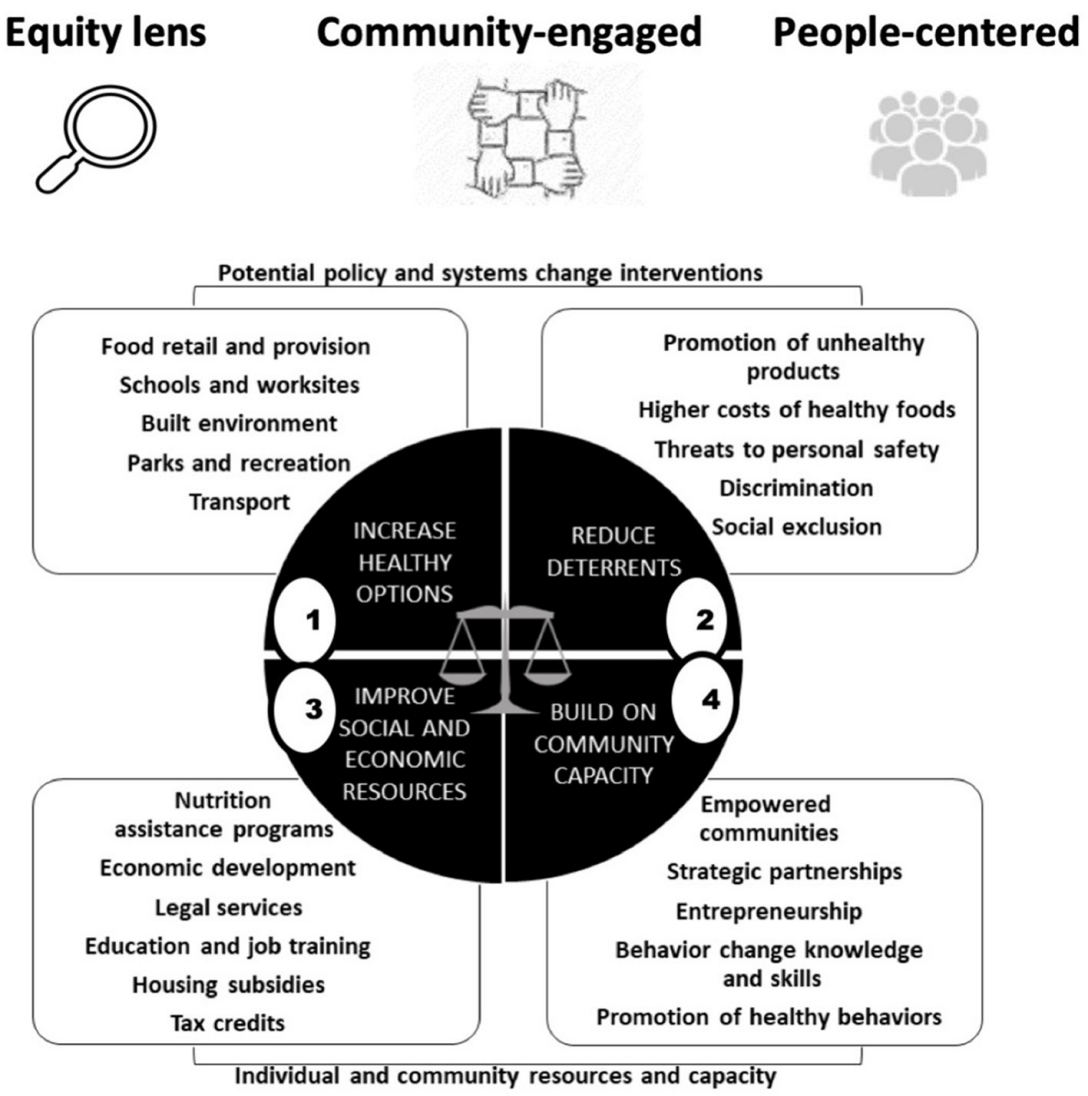
GTE framework graphic: four sets of potentially complementary considerations for identifying equity considerations in policy, systems, and environmental change approaches. Source: adapted from Kumanyika (5).

The Getting to Equity in Obesity Prevention Framework (GTE) was specifically developed to aid researchers and practitioners in understanding and operationalizing equity considerations in PSE interventions (5). The framework references expert recommendations about the types of changes needed to improve the impact of obesity prevention efforts generally (2, 4). It proposes a process for undertaking intentional efforts to address equity in such work by prompting for identifying relevant design, implementation, evaluation and analysis considerations related to equity impact. The guidance—a two-dimensional, circular logic model (Figure 1)—divides relevant considerations into four quadrants: (1) PSE approaches to improve healthy options; (2) deterrents to the effectiveness of those approaches; (3) improving individuals' social and economic resources; (4) and building on community capacity. An icon representing the anticipated synergy that is at the core of how equity interventions operate (16), i.e., a systems perspective, is superimposed in the middle of the circle (see Methods for a further description of the GTE framework). Several published articles apply the guidance in the four quadrants in some way (17–23) but to date no applications include operationalization of the systems perspective by analyzing interactions among domains of the GTE.

Systems thinking and systems science approaches can enable understanding of complex relationships fundamental to the intent of the GTE framework (16, 24). A growing body of work has applied specific systems methods such as systems mapping, causal loop diagraming, and system dynamics to equity-focused obesity efforts (25–28), but most are calibrated to their specific study context and not applied with the specific intent of enhancing the usability of an existing and robust equity framework. Building on existing work, the overall objective of this study was to illustrate ways that pairing the equity framework with systems mapping could provide insights into complex, non-linear relationships not otherwise visible from a framework with a linear theory of change. We conducted a retrospective, secondary analysis of data from the Childhood Obesity Declines Project (CODP)—a case study of various concurrent and partially coordinated set of initiatives active in Philadelphia, PA before and during 2006–2010. Aims were to (1) examine how and in what ways the PSE interventions identified as most critical to observed reductions in childhood obesity prevalence worked together (i.e., explicitly illustrate synergistic interactions), and (2) draw attention to certain indirect or unplanned effects of the interventions, and how these emergent effects influenced the overall ability of the system to achieve its goal.

## 2 Materials and methods

### 2.1 Study design

The Philadelphia case study was one of four such childhood obesity prevention studies commissioned by the National Collaborative on Childhood Obesity Research (NCCOR) from 2006 to 2010 to identify possible contributors to declines in childhood obesity (29, 30).^3^ We used systems mapping to incorporate systems thinking perspectives into the use of the GTE framework to more explicitly capture reinforcing synergistic effects and better illustrate how, in a multi-intervention context, any one strategy might impact a subsystem embedded within the web of factors interacting to facilitate the childhood obesity declines in Philadelphia. Being able to illustrate dynamic interactions, or feedback, within a theory of change can be helpful for *post hoc* analysis and, by extension, intervention planning and implementation.

### 2.2 Data source: Philadelphia Childhood Obesity Declines Project (CODP)

The study context, methods and findings of the Philadelphia CODP are available from a detailed report on the NCCOR website, supplemented by a series of articles providing further methodological details, analyses, and perspectives (31–33). Research sites for CODP were selected on the basis of reports of statistically significant declines in child obesity prevalence associated with concurrent or recent community-wide initiatives. The Philadelphia site, with ~190,000 enrolled 5- to 18-year-old students in the School District of Philadelphia (SDP)—the eighth largest school district in the U.S.—was highly relevant from a health equity/health disparities perspective. When the baseline data were collected in 2006–2007, the majority of students in the SDP were African American (62.9%) or Hispanic (16.6%), with nearly half from families eligible for free and reduced-price school meals (48.9%) (34). These percentages were similar or, for free and reduced priced meal eligibility, higher (57.4%) among SDP students in 2009–2010. National data for the period between 2006 and 2009 indicated higher prevalence of obesity among children and adolescents in the non-Hispanic Black and Hispanic categories compared to the non-Hispanic White category. These disparities were observed in one or both sexes overall and in some cases when stratified by low-, middle, and high-income (13, 35).

Obesity prevalence across the CODP study period was estimated from annual height and weight measurements collected for about 60% of the SDP students (similar but varied from year to year) with a similar demographic breakdown to the overall SDP population. Overall, in the data for children in priority populations, childhood obesity and risk reduction declines in prevalence were small but statistically significant, a 4.8% relative decrease for obesity (21.5%−20.5%; *p* < 0.001) overall and a 7.7% decrease (8.5%−7.9%; *p* < 0.001) for severe obesity, and were significant for Hispanic and African American children in one or both sexes and for obesity in non-Hispanic white males (34).

### 2.3 Data extraction

The CODP research approach used an adaptation of the Systematic Screening and Assessment methodology (36) to collect narrative data including key informant interviews about PSE relevant initiatives in the Philadelphia community during the pre-study period beginning as early as 1991 through the period of data collection between 2006–2007 and 2009–2010. The narratives described both the initiatives and how they were implemented. The matrix of strategies identified in the main report included 30 policies and programs (29). For the present analysis, data were extracted for the four nutrition-related strategies that key informants interviewed for the case study viewed as having been focal in contributing to the obesity declines—likely to have reached both the largest number of children and the children at highest obesity risk. These strategies occurred within or were linked to the schools concurrent with the CODP study time frame: a Universal Feeding Pilot (UFP) for School Meals; a policy, Eat Right Now, that provided for nutrition education delivered in both school and community settings; a ban on sugary drinks on school premises; and a comprehensive, district-wide School Wellness Policy. Extracted details for these strategies are provided in Supplementary Table 1. The descriptions included details of approaches, barriers, facilitators and partner roles that permitted inferences about pathways through which the interventions were linked and supported the systems mapping. We also consulted a government report on the early experience with the UFP for additional clarity about how the Philadelphia SDP administered and implemented the program (37).

### 2.4 The “Getting to Equity in Obesity Prevention” Framework (GTE)

Shown graphically in Figure 1, the icons at the top of the framework represent foundational principles for applying the program using an equity-sensitive perspective—an “equity lens,” using a community-engaged approach, and maintaining a “people-centered perspective” that considers the socioeconomic and sociocultural circumstances, values, priorities and needs of the people in the settings or environments of interest. The circle in the center has four quadrants with examples of possible associated actions or equity-related aspects in the callouts for each quadrant. Numbering of the quadrants is for ease of reference, although the intended use of the framework is iterative and not necessarily sequential. The top two quadrants relate specifically to PSE change approaches. Quadrant 1 focuses on a main policy or programmatic change of interest and its relevance to an equity focus on the setting and population. Quadrant 2 prompts for factors that might work against the effectiveness of this approach in the setting and population of interest. The bottom two quadrants relate to enhancing individual (Quadrant 3—facilitating resources that can assist with social and economic needs linked to participating in and benefitting from the intervention) and community resources and capacity (Quadrant 4—identifying and enhancing community level assets, health promotion contexts, and resources to foster uptake and sustainability). Most pertinent here, the scales of justice in the center prompt for systems thinking, underscoring the potential for synergistic effects on equity from a combination of multidimensional, mutually reinforcing elements in the four quadrants. A more detailed explication of the GTE framework is available as an online user toolkit (38).

### 2.5 Systems perspectives for interventions

Logic models are often the tool of choice in illustrating the types of multifactorial intervention processes that would be modeled using the GTE framework, which requires inputs from multiple domains and different types of activities to achieve a desired outcome. They can represent the sequential set of inputs, activities, outputs, and outcomes that need to cumulatively occur in order to achieve the desired goal or outcome (39, 40). For example, activities across GTE quadrants 1 and 2 can be viewed as operating on an additive scale, that is: the combined impact of policy or programmatic change and the factors working against that change can be understood through traditional linear approaches (i.e., actions to improve obesity minus deterrents that operate counter to those actions equal the net intervention impact). However, the linear theory of change presented in logic models is limited in capturing synergies between activities or actions that emerge through feedback relationships between factors (39, 40). Feedback or mutually reinforcing processes, such as those capturing the multidimensional activities in quadrants 3 and 4 of the GTE framework, require tools that can illustrate cumulative, non-linear effects, and the ways in which they amplify impacts on desired change. These activities occur in a context that can better be understood as a system.^4^ Box 1 details the key terminology used to describe systems and their properties. Within a systems lens, the non-linear effects characteristic of quadrants 3 and 4 are produced by the pattern of interrelationships, feedback, and time delays that accumulate to produce a particular behavior over time (see Box 1). In this case, the system is the set of actions that can lead to equity in the impact of addressing childhood obesity. Within the systems toolkit, which includes a wide array of approaches to analyzing systems (42, 43), we chose a systems mapping approach because of its detailed visualization tools. These tools are helpful in illustrating the specific inputs, activities, outputs, and outcomes, similar to logic model diagrams, but also to visualize interrelationships between factors and with contextual variables, such as community and social context that characterize the scales of justice at the center of the GTE framework. The diagrammatic visualizations from systems mapping are helpful for facilitating shared understanding and action planning.

Box 1 **Definition of a system and its properties.**

*System:* a set of interrelated elements that interact to achieve an inherent or ascribed purpose.

*Interrelationships:* the way in which system elements interact or work together to generate a desired outcome. Failure to account for interrelationships can prevent insights into how the system produces the desired outcome or, alternatively, unanticipated behaviors.

*Feedback:* the constant, changing behavior of systems is a response to both interrelationships between factors and the history of behavior experienced and/or observed to date. Systems are often characterized by feedback behavior through which they adjust future actions based on previous actions.

*Time delays:* the effect of one element of the system on another may not be instantaneous; it may lag behind its cause due to “accumulations,” or the stockpiling, of elements in places along the causal path. This can result in unexpected or hard to predict behavior over time.

*Boundaries:* clear statements around the scope needed or applied when understanding or acting to address a problem at hand. Negotiating the boundaries needed to understand, act, and implement solutions within a system is critical to setting realistic expectations and creating collaborative networks to act on the right leverage points.

*Multiple perspectives:* different actors or stakeholders operate from different positions within a system and are needed to effectively understand and negotiate the aforementioned four characteristics of systems discussed here.

Source: Meadows and Wright (41).

### 2.6 Analytic framework

The GTE framework guided analyses in which selected observations from the CODP data for Philadelphia were classified according to the GTE quadrants (see Section 2.4), and then conceptualized from a dynamic systems perspective using causal loop diagramming (CLD)—a qualitative systems mapping approach—to illustrate how the multi-component strategies undertaken in Philadelphia during the study period might have interacted to lead to the observed declines in childhood obesity (see Section 2.2). We use CLDs because they are a systems mapping approach that provide a useful balance of visualizing not only the elements in the system (i.e., variables) but also how they work together (i.e., causal connections, direction of causality, delays) while remaining largely accessible to public health audiences.

**Figure 2:**
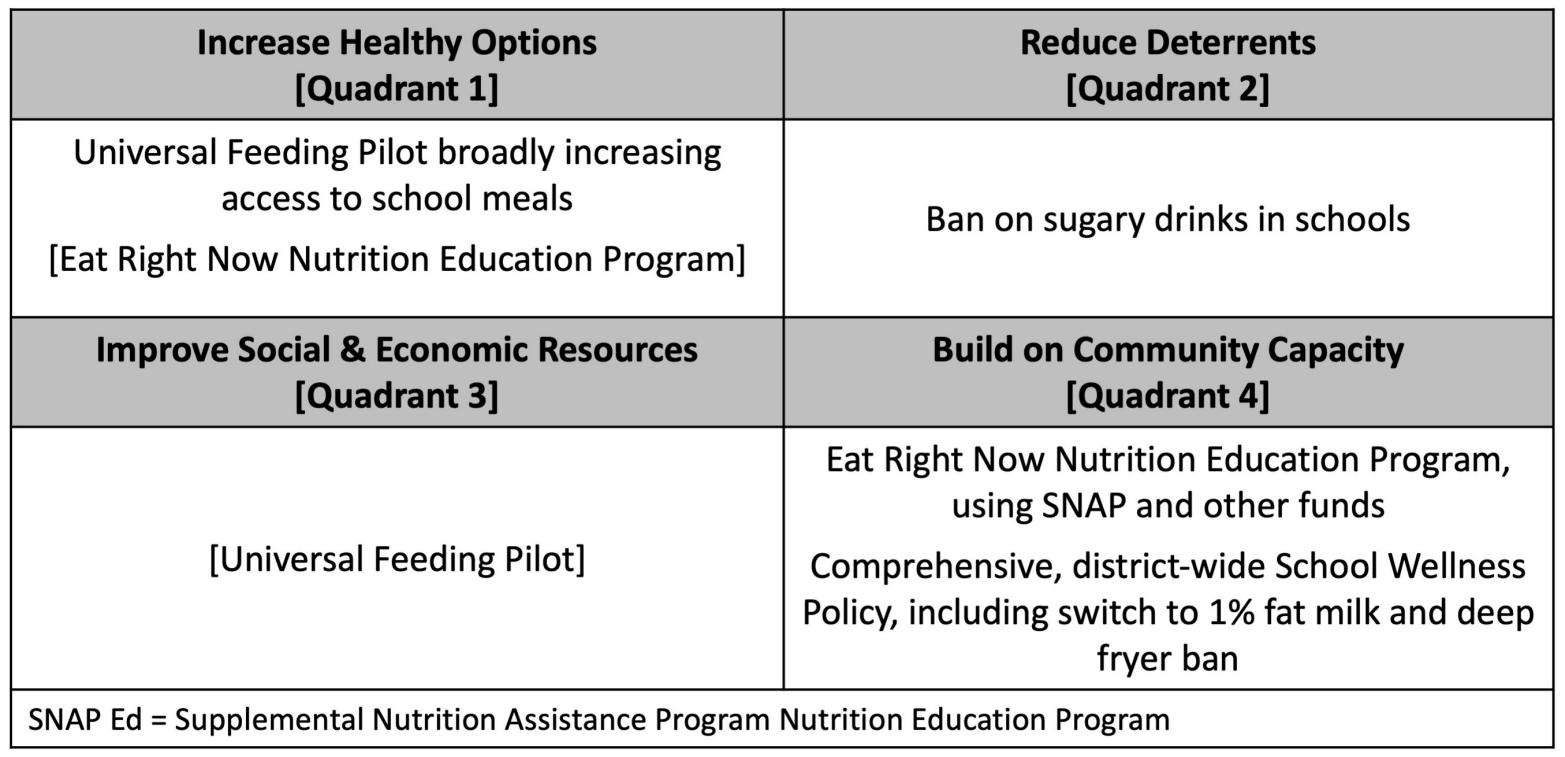
Mapping the Childhood Obesity Declines Project (CODP) focal initiatives onto the Getting to Equity (GTE) Framework's quadrants.

For the initial GTE-based systems mapping, we focused on the four interventions discussed in Section 2.3 that focused on increasing access to and consumption of healthier foods and beverages in schools (see Figure 2). The Universal Feeding Pilot was mapped to Quadrant 1 but was also recognized as relevant to Quadrant 3 because the program intrinsically provided financial benefits to families, in addition to its main goals of addressing food insecurity and improving access to healthy food options through meals. The SNAP Ed “Eat Right Now” program was mapped to Quadrant 4 because it was interpreted as reaching the broader community, but was also mapped to Quadrant 1 as a school-based educational program.

**Figure 3:**
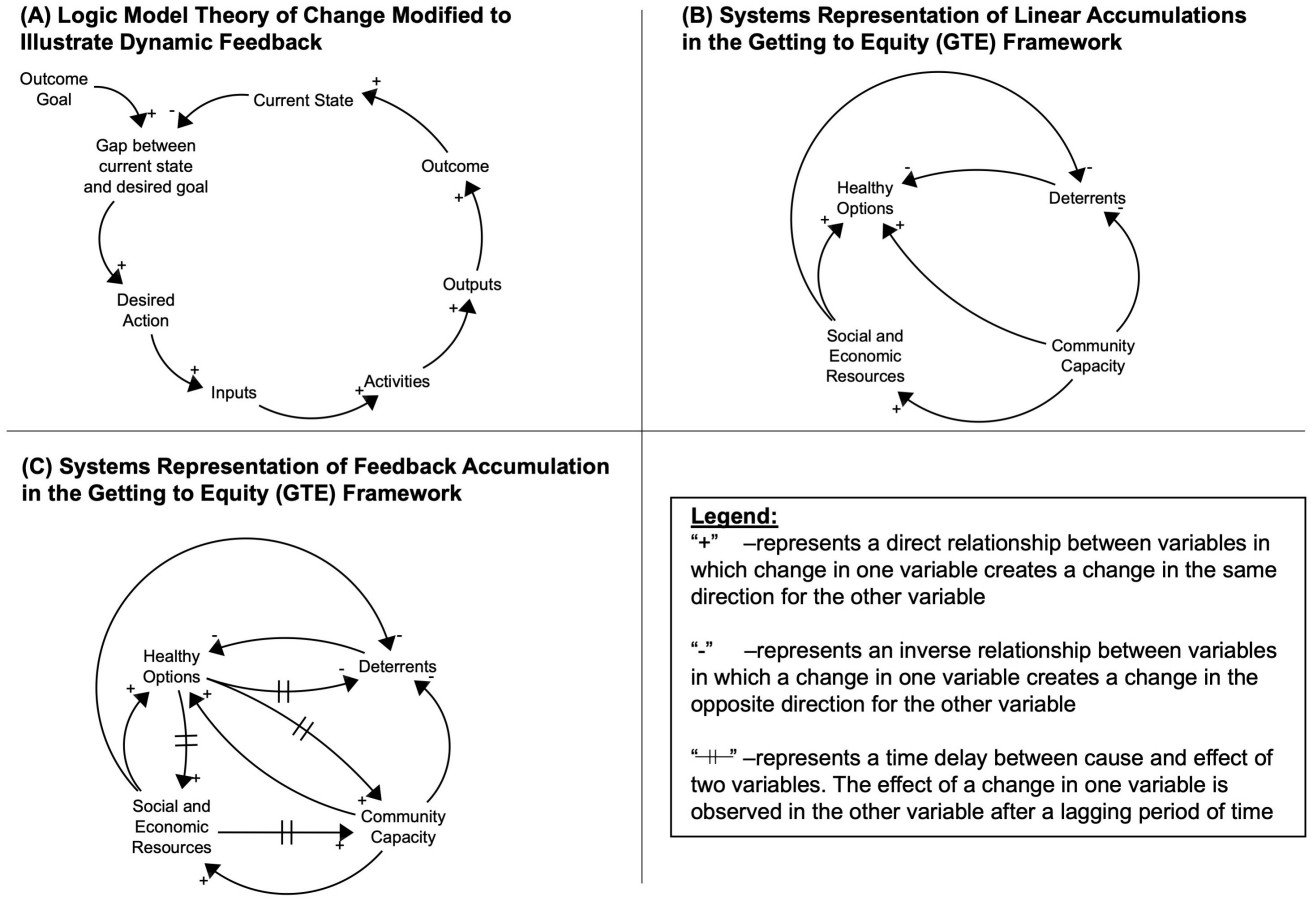
General systems feedback structure for interventions **(A)**, and generalized systems map for the Getting to Equity (GTE) Framework **(B, C)**.

Figure 3 shows the generalized model used to anchor our systems analysis of the CODP case study, developed from standard system structures (39, 44). The model starts by defining the intervention target in a goal/gap structure where the current state of the outcome of interest differs from the desired goal of that outcome creating a gap that leads to action being taken (often in the form of an intervention; Figure 3A). That intervention then develops inputs, activities, outputs, and outcomes to influence the current state such that it grows closer to the desired state. In Figures 3B, C, we use the same systems diagramming approach to represent the GTE framework; successively building out the reinforcing feedback pathways that link the quadrants together. We first created basic connections between the quadrants of the framework that reflected their descriptions (Figure 3B). Next, we add in the synergistic reinforcing relationships between the quadrants (Figure 3C). This format also allows us to represent the long-term time delay (illustrated by the hash marks on the arrows representing an accumulation) between the cause and effect impacts of successful interventions to promote healthy options. In particular, as the intervention is successful and shifts the current state of obesity to the ideal state of obesity, the deterrents are then less influential, and there is more capacity to improve other social and economic resources and build on community capacity to address increases in obesity prevalence. Additionally, the social and economic resources that families accumulate over time, allow them to contribute to the community's capacity to respond to obesity reduction strategies and activities. Using the three systems diagrams as our analytic heuristic, we applied them to the CODP case study to depict the contributions that systems mapping can add to understanding success in a dynamic, equity-oriented context.

### 2.7 Systems mapping approach

We used causal loop diagramming, building on the core feedback framework for intervention and program planning or—in this analysis, retrospective—evaluation, illustrated in Figure 3 to, analyze the case study described in Section 2.3. We first illustrated reinforcing feedback in the core GTE model as an anchor for synthesizing activities and factors present in the case study. Then, using the core heuristic in Figure 3, we built in the CODP-specific activities, factors, and interrelationships that were identified as driving successful outcomes in Section 2.2.

The four focal initiatives defined the boundary of our analysis around initiatives that occurred in the same/similar contexts, i.e., were school related. We extracted details about the four focal initiatives (see Supplementary Table 1) and iteratively reviewed the strategy descriptions to identify variables or factors reported as part of the success of each strategy, the causal connections between factors, and the direction of these causal relationships. We assessed whether a change in the causal variable created a change in the effect that went in the same direction (positive relationship) or whether a change in the causal variable created a change in the effect in the opposite direction (negative relationship).

After identifying variables, causal connections, and the direction of the causal connections, we used Vensim software version 10.1.4 (Ventana Systems, Inc., Harvard, MA) to create a diagram detailing the systems map. After mapping these connections, we used causal loop diagram guidelines to identify feedback loops throughout the causal connections and assess whether these feedback loops were reinforcing—creating more of the behavior that the loop created in the first place—or balancing—counteracting the behavior that the loop created in the first place. We also classified variables and loops based on the quadrants described in Figure 2 and coded them with different line patterns accordingly: thin solid lines for increasing healthy options, thick dashed lines for reducing deterrents, thin dashed lines for improving social and economic resources, and thick solid lines for building on community capacity. As a research team, we verified our interpretations and adjudicated any differences through discussion within the team, which included drawing on direct observations and subjective insights from the team member (SK) who was aware of and in some case involved in the childhood obesity prevention efforts during the pre-study and active study periods. Through these discussions, we identified an additional important system process that was not explicitly represented in the GTE model, and integral to the success of each initiative; we identified unintended consequences and co-benefits as key parts of system structure and used a dotted line to classify them. Throughout this process, we iteratively refined the map of the system for both parsimony and accuracy.

## 3 Results

**Figure 4:**
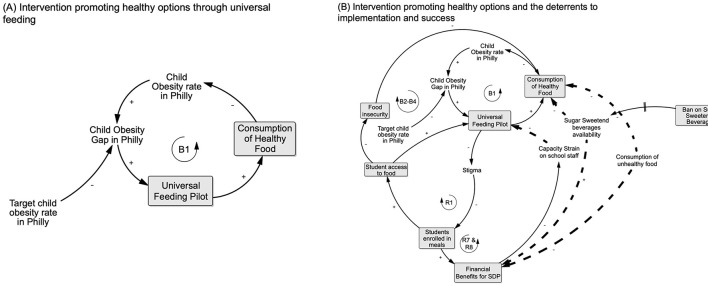
Intervening on obesity: quadrant 1 starting in **(A)** depicts the overall process (B1) whereby the Universal Feeding Pilot (UFP) contributed to closing the gap between the observed vs. desired, lower prevalence of child obesity (see explanation in text). **(B)** Adds detail relevant to Quadrant 1 by showing the cumulative processes through which the UFP introduced school-wide access to and consumption of healthy food: removing eligibility-related barriers to participation (B2–B4), reducing poverty-related stigma associated with school meals (R1) which in turn increased student participation, food access, and reduced food insecurity (B2–B4). Increased student participation was associated with financial benefits for schools (R7) as well. Quadrant 2 adds processes that reduced deterrents as potential challenges to effectiveness are also shown (R7; see text for explanation).

**Figure 5:**
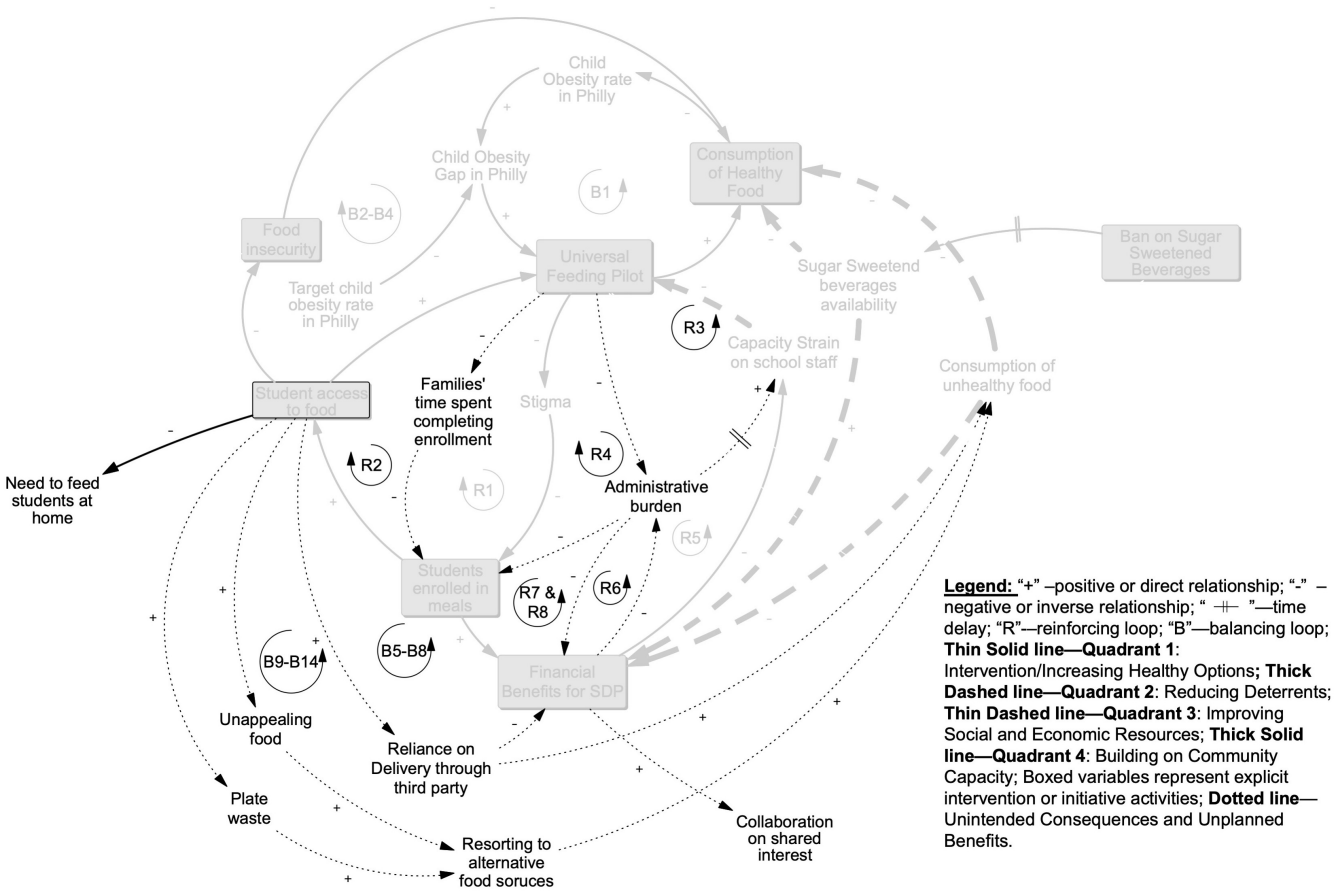
Unintended consequences and positive side effects: processes depicted in this picture emerged from implementing intervention activities, but did not fall within a particular quadrant. As a result of the UFP providing school-wide access to meals, positive side effects included families saving time on having to complete individual enrollment which increased enrollment in the program (R2), reducing the administrative burden of implementing the UFP on the schools end (R4), and increasing the financial benefits (R6) which worked to alleviate capacity strain on school staff (R3 and R7&8). Implementing the UFP was not without its unintended consequences, with unappealing food and plate waste both leading students to resort to other, less healthy food sources (B9-B14) and schools without kitchens having to rely on third party deliveries which both cut into financial benefits as well as increased consumption of unhealthy food (B5–8) (see explanation in text).

**Figure 6:**
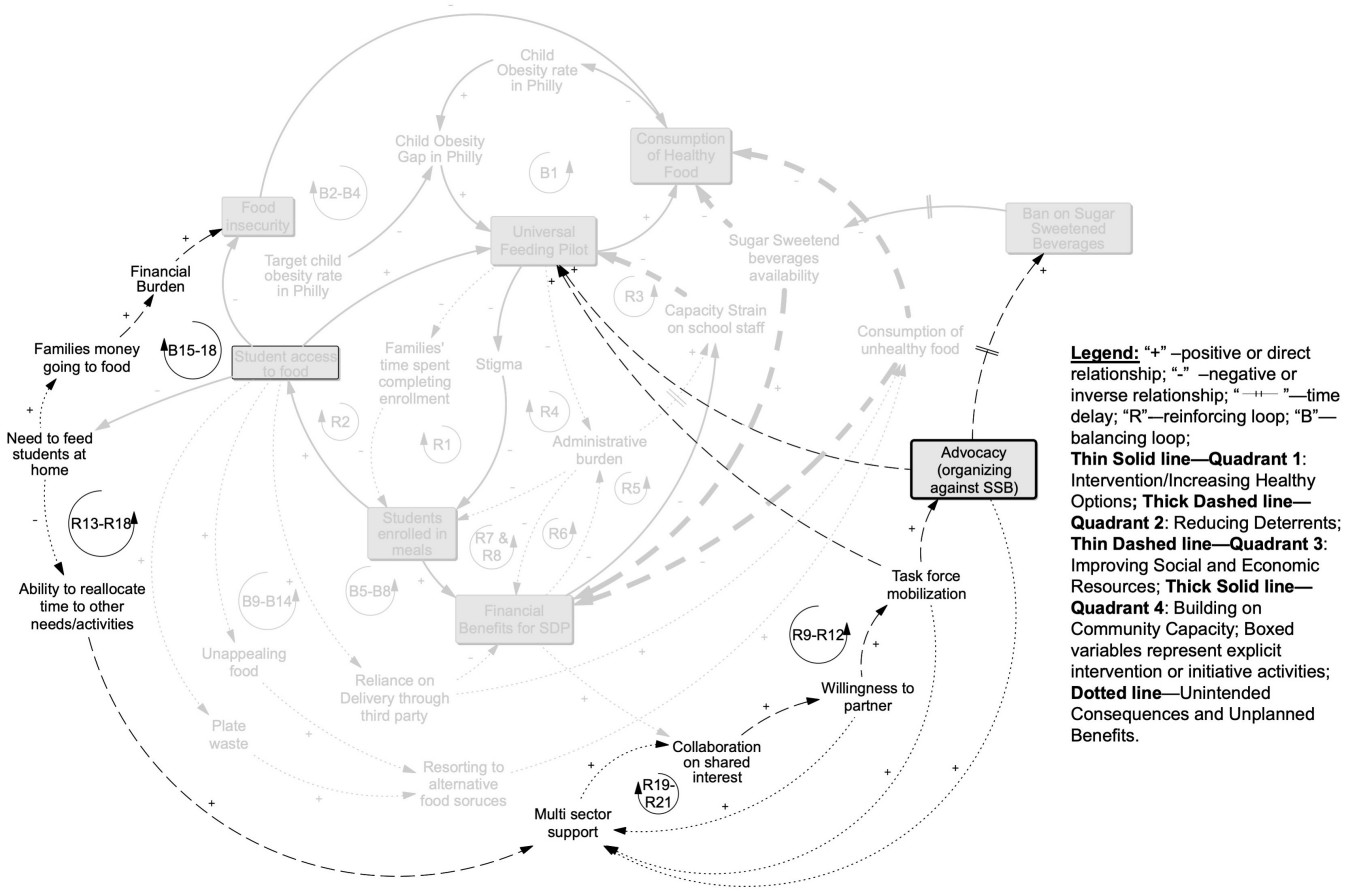
Quadrant 3 increasing social and economic resources: benefits from the UFP increasing access to food we assessed as reducing families need to feed students at home which decreased the money going to food and alleviated the financial burden of families while reducing food insecurity and increasing healthy food consumption (B15–B18). Families were also purported to have increased ability to direct time to other activities (R13–R18). This was anticipated to have an impact on multisector support which in turn increased collaboration of partners on shared goals (R19–R21) and through this willingness to partner, they were able to mobilize to both to protect the UFP from being cut by USDA and advocate for a larger sugar sweetened beverage ban, thus further decreasing the deterrents from healthy eating and the UFP (R9–R12; see further explanation in text).

**Figure 7:**
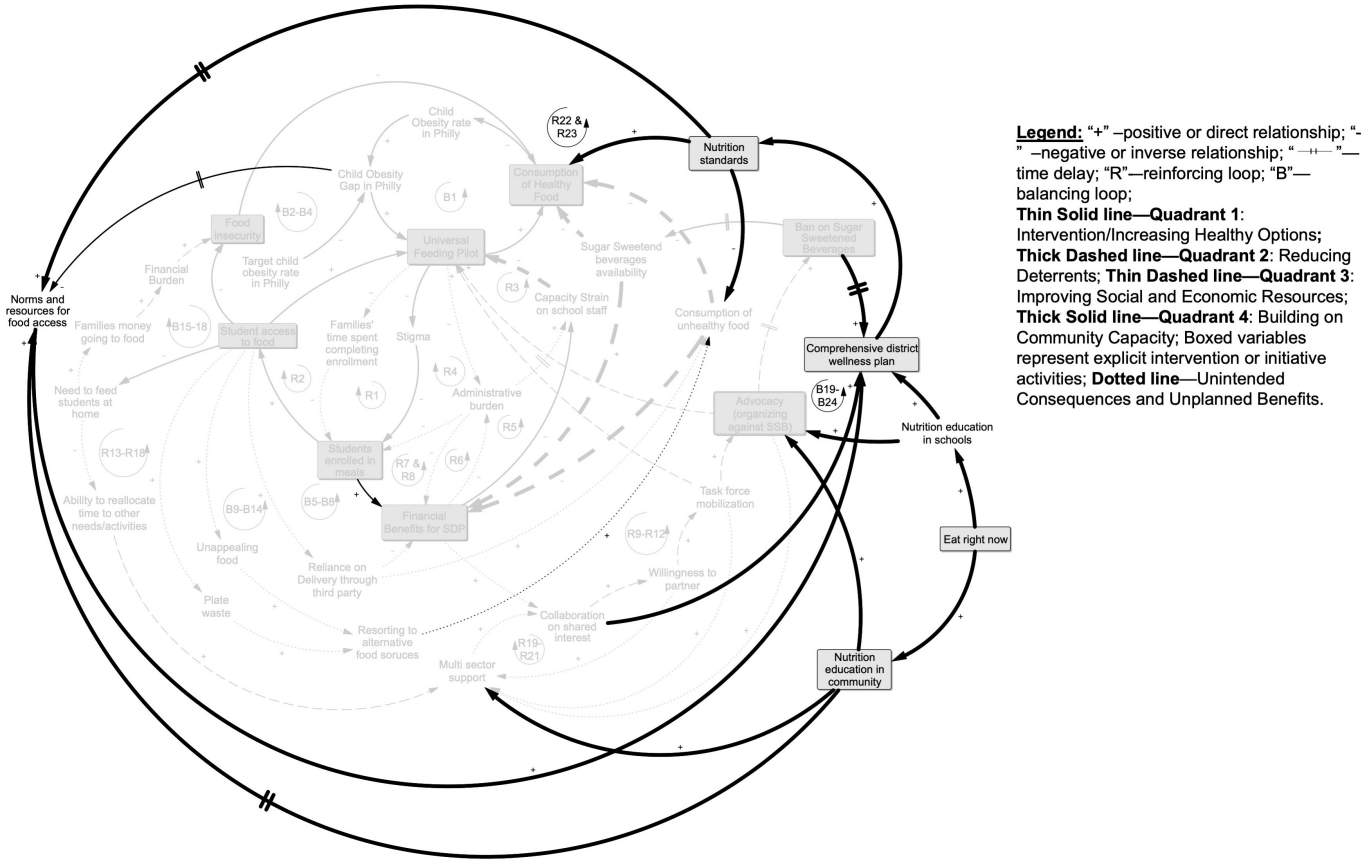
Quadrant 4 building community capacity: the ongoing Eat Right Now initiative operated in schools and the community to increase nutrition education and along with the outcomes of collaboration led to the comprehensive district wellness plan, which reinforced healthy eating standards district-wide (B19–B24). Over time the Comprehensive District Wellness plan shifted nutrition standards in ways that shifted the norms around school nutrition and the resources available to promote a wider set of obesity reduction activities across the city (R22&R23; see text for further explanation).

**Figure 8:**
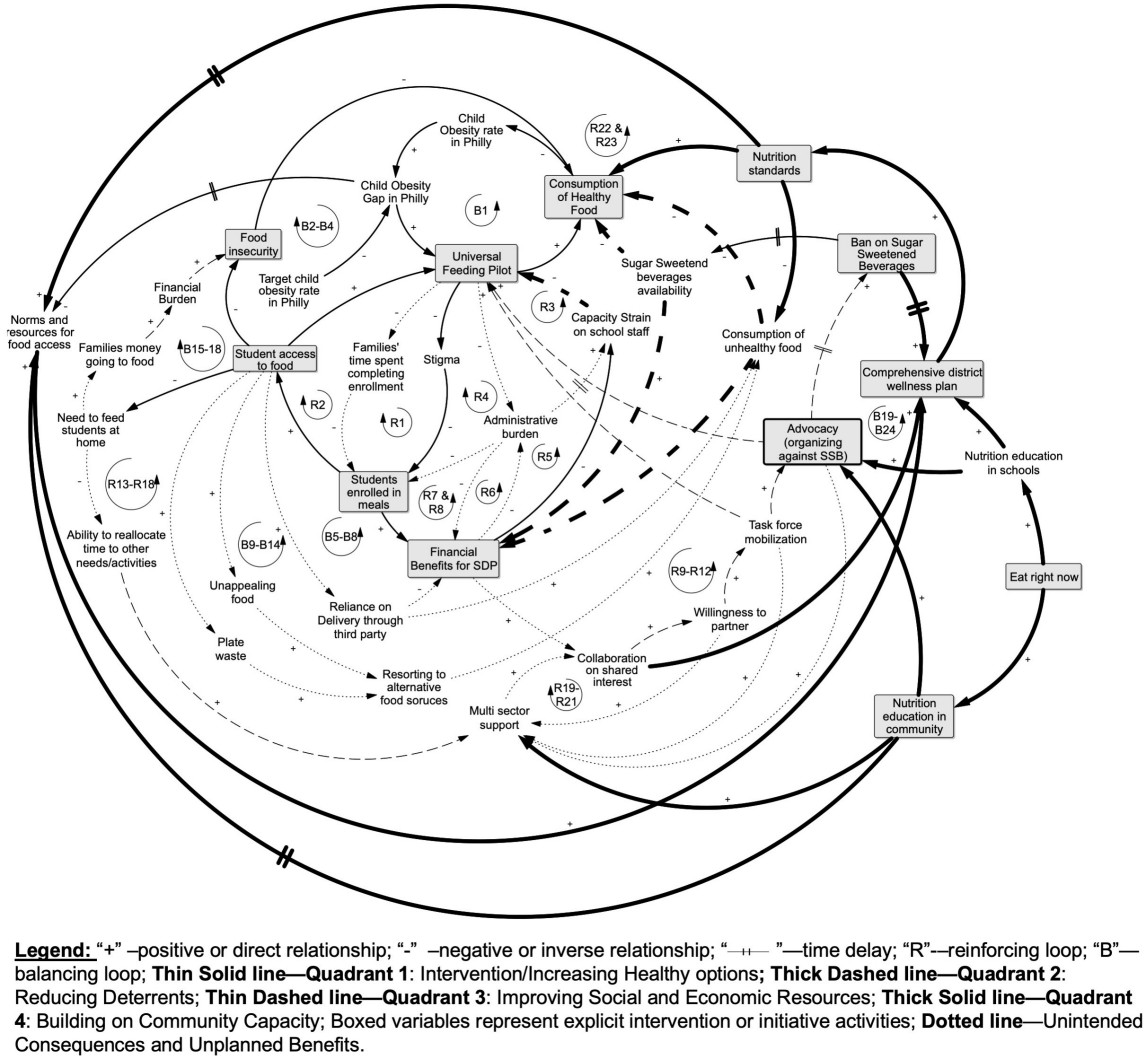
Full system map of the GTE quadrants applied to CODP Interventions to address childhood obesity in Philadelphia.

Our systems map depicts four interconnected subsystems corresponding to the four quadrants of the GTE framework with the additional characterization of unintended consequences and co-benefits we identified or posited (Figures 4–8) as connecting or emerging from the quadrant subsystems based on a combination of information in the Philadelphia CODP report and our analytic process. The final systems map included 47 individual loops, 23 reinforcing loops, and 24 balancing loops, which we reduced to 17 consolidated key loop structures. We consolidated individual loops for parsimony; individual loops were combined if they illustrated the same loop behavior with only slight variations in pathways to achieve it. Next, we provide a detailed description of system structure based on the quadrants of the GTE Framework. Items outlined in boxes in the figures were explicit intervention or initiative activities, whereas elements not in boxes were factors that we identified through our analysis as key to system structure and function. Individual loop descriptions are available in Supplementary Table 2.

### 3.1 Increasing healthy options (Quadrant 1)

In Quadrant 1 (Figure 4A), loop B1 represents the system structure of the intervention that CODP identified as improving healthy food options for Philadelphia public school children. The UFP was implemented to address food insecurity, as illustrated in loop B2 in Figure 4B, but was also expected to improve children's healthy food consumption because of the nutrition standards for school meals and other foods available in school environments and the concurrent nutrition education program which are illustrated in depth in Quadrant 4. Increasing access to healthy food for a substantial proportion of public-school children could potentially contribute to closing the gap between observed and targeted obesity prevalence in this population. Stigma associated with having a free- or reduced-price meal was identified previously as a barrier to participation in the National School Meals program when individual children qualified based on a poverty-threshold. The UFP addressed this potential deterrent, as indicated in loop R1, to program participation by shifting eligibility to the school- rather than individual child level. Making all children in a qualifying school eligible to receive free school meals avoided the need to stigmatize some children as “poor” and increased participation. In turn, the increased participation, led to higher “tray” counts which provided direct financial benefits for the SDP as illustrated in loop R7, based on the per meal reimbursement rate.

### 3.2 Reducing deterrents (Quadrant 2)

The objective in Quadrant 2 is to identify and reduce deterrents to effectiveness of the intervention of interest. Figure 4B shows the capacity strain that was present for school staff before the UFP pilot. They were required to enroll students in the School Meals program individually and manage individual families' applications. Staff had to review paperwork, manage eligibility criteria, and process applications on a constant basis since eligibility status was in flux with most families. These capacity strain factors were alleviated, as illustrated in loop R7, due to the pilot's school-wide eligibility and streamlined process of counting and tracking the number of meals served. At the start of the UFP, an additional challenge was the availability of sugar-sweetened beverages (SSBs) in some schools through vending machines. These vending machines had unhealthy food options that students could access if they found the vending options more appealing or palatable than the food served in the school meals. However, an intervention to remove SSBs from vending machines in some schools was implemented prior to the implementation of the UFP in anticipation of this being a detrimental deterrent.

### 3.3 Unintended consequences and positive side effects (co-benefits)

Our analyses identified several unintended adverse consequences and positive side effects (co-benefits) that, in retrospect, provided some of the bigger challenges or were integral to UFP success (Figure 5). In particular, there were three main positive side effects that built up to subsequent greater gains for later program implementation. Loop R2 depicts families' benefit by: (1) spending less time completing enrollment; (2) having the option of spending less time in food preparation; and (3) having more money to spend on food for other purposes or other things when their children ate school breakfast and lunch. As we will explore more in Quadrant 3, we interpreted this as having important implications for families' ability to improve their social and economic resources as well.

On the school side, the removal of responsibility for identifying and differentiating children who were eligible for a free or reduced-price meal vs. paying for meals or tracking and collecting lunch payments alleviated administrative burden and was a major positive side effect of the UFP. Also, rolling out a meals program on a larger scale was anticipated to increase capacity strain but this was mitigated by the greater saving of staff time, as described in loop R3, as well as the potential for increased funding associated with more students taking school meals, as depicted in R6 and R8.

However, there were challenges associated with three unintended consequences. First, while food was more available through meals, as explored in loops B9–14, students reported that the food was unappealing and thus they still relied on alternative, often unhealthy, food sources during the day rather than taking the school meal. These sources included corner stores near the schools, which were a known deterrent to healthy eating even before UFP implementation. A concurrent pilot program that targeted corner stores near schools to improve food options and choices (through in-store education) served as a counterbalance to this challenge. Second, for students who did take the school meal but found it unappealing, both not consuming the school meal (i.e., plate waste) and reverting to unhealthy foods were problematic, as demonstrated by the processes in loops B9-B14. The SDP would count these as reimbursable meals but the program objective of students actually consuming healthier meals would not be achieved in those cases—resulting in a waste of program resources. The third challenge was that most schools did not have the in-house capacity to cook and/or serve meals. This increased the reliance on food delivery that was not always healthy or cost effective through third party vendors and would also have impacted the meal quality, as depicted in loops B5–B8.

### 3.4 Increasing social and economic resources (Quadrant 3)

Interventions in both Quadrants 1 and 2 and the positive side effects discussed above set the stage for amplifying the social and economic resources that families could leverage (Figure 6). In particular, because families did not need to feed students at home, they both had more money for other financial needs (loops B15–18) and more time to attend to other things (loops R13–18). This positive side benefit for families aligned with the objectives of the partners who saw the UFP as a way to address poverty and food insecurity. It led to an additional, positive co-benefit of increasing multisector support for food and nutrition programming in communities, depicted in loops R13–R18, and collaboration between stakeholders that enhanced social capital through willingness to partner (loops R19–R21) and ability to mobilize. In turn, the enhanced willingness to partner further amplified the ability to advocate for additional policies, particularly supporting the SSB ban district-wide as depicted in loops R9–R12. Furthermore, social capital gains fed back on themselves to amplify the multi-sector support.

### 3.5 Building on community capacity (Quadrant 4)

The accumulated gains from the co-benefits and the social and economic resource improvement both impacted and were further amplified by community capacity enhancement efforts through a few key programs (Figure 7). The Eat Right Now program was active in, but not limited to, schools; it reached children and adults in the Philadelphia community at large. This education, combined with collaboration gains discussed above set the stage for the Comprehensive School Wellness Policy which, in turn, impacted the nutrition standards overall, as we illustrate in loops B19–B24. Finally, over time, these efforts to leverage community assets and enhance capacity fed back to influence norms and resources for food access that further shaped the foundation from which food and nutrition policy and broader health promotion efforts were approached going forward and potentially accelerated the trajectory toward closing the child obesity gap in Philadelphia, which we illustrate in loops R22 and R23.

### 3.6 Full systems map

Figure 8 presents the full systems map for the CODP assessment of how the four focal interventions in Figure 2 intersected to result in cumulative, dynamic processes. This map illustrates how retrospectively operationalizing the synergistic and mutually reinforcing pathways using systems mapping provided a practical way to understand the cumulative processes that had to operate in concert to achieve even a modest gain in obesity reduction among public school children in Philadelphia. Furthermore, by clearly illustrating actions from each quadrant, we illustrate that factors across quadrants do not have to be balanced in number in order to result in equitable outcomes.

## 4 Discussion

In this analysis, we used a systems mapping approach to expand upon the synergistic element of the GTE framework. We focused on the GTE framework because it was developed to address equity issues in obesity and related health issues, and explicitly includes the principle that combining interventions across and among the four quadrants will be more than additive—indicating the potential for synergy with the scales of justice in the center. However, current guidance for applying the framework has not yet included formal use of systems science tools, and this study addresses that gap. Our goal to illustrate the value added by using systems mapping tools with a GTE framework application was motivated by the increasing awareness that PSE initiatives intended to improve population- and community-level contributors to obesity prevention are embedded in complex, dynamic systems, along with evidence that systems thinking and the use of systems science tools can enable characterization of these systems and guide identification of points of leverage for positive change (16, 24). The systems mapping and causal loop diagrams were selected from the wide away of available systems science tools (42, 43) based on the fit of this approach with visualizing processes integral to how the GTE framework can support equity in PSE approaches for childhood obesity prevention.

This case study based on the Philadelphia CODP was a good example because it focused on children at high risk of obesity who were disproportionately low-income and racial/ethnic minoritized populations and involved a set of programs with a wide reach and a shared goal of increasing access to and consumption of healthy foods with potential for sustainable change at the population level. A prior application of the GTE framework analyzed a comprehensive healthy school program and discussed the potential for synergy on an intuitive basis (22). Here, we were able to move beyond singularly understanding the UFP as the main, focal intervention to visualizing and understanding synergy through interconnections between the UFP and other concurrent, school-related programs that were associated with the observed declines in obesity prevalence in Philadelphia, and in some subgroups of African American and Hispanic children.

One of the key strengths of taking a systems approach to understanding complex problems is the ability to work with and understand the implication of time scales. The use of time scales has been illustrated across a wide array of contexts from assessing the behavior over time of key trends impacting healthy eating across 49 sites in a large multisite project (26) to modeling a more detailed understanding of neighborhoods as a dynamic determinant of health inequities during key windows of the life course (45). In the context of our case study, being able to visualize this dynamic process of community capacity building, which occurred over a longer time scale during both the pre- and active CODP study period, using systems tools contributed a deeper level of appreciation for the history or foundation needed for the fourth quadrant of the GTE framework to optimally contribute to program success. The CODP accounts of actions that occurred in the pre-study period was advantageous in this respect. Accumulated community capacity that was built over time and accelerated by implementation of the UFP program, e.g., leading to collaboration targets for stakeholders to align with, was integral to its success.

Our case study demonstrates the challenge of boundary setting that is intrinsic to applications of systems approaches and ended up, in this case, being iterative to allow inclusion of initiatives that were found to be relevant. However, when used for either retrospective evaluation or prospective planning, setting boundaries for a systems map requires threading a needle between being parsimonious while still being able to visualize enough detail of the system to understand why desired or undesired behaviors arise over time. In this analysis, when developing our system map, we first set the boundary to include activities that were directly related to and proximal to the school setting in which students were experiencing the UFP. The concurrently occurring Healthy Corner Store Initiative (29), which was being piloted in stores near schools to increase access to and motivate consumer selection of healthier food options, was assumed to be outside of the boundary of interest. As the analysis evolved, it became apparent that two of the main unintended consequences of the UFP, plate waste and unappealing foods related to students not taking or consuming school meals, were operating to impact unhealthy food consumption through the presence of corner stores with unhealthy food options. As such, the Healthy Corner Store Initiative pilot to decrease these deterrents was actually embedded within the system driving the school-based initiative's success.

Finally, while our case study is retrospective, we emphasize the applicability and importance of using systems mapping or other systems thinking tools, including participatory and community-based approaches (46–48), early in and during GTE-informed program planning as well as forensic analysis/*post-hoc* assessment. Systems maps can be strong complements to logic models often used to illustrate theories of change at this stage and can be living documents that evolve as the interactions between interventions unfold over time and are better understood. For example, Owen et al. used a community-based system dynamics (CBSD) approach to understand, retrospectively, the pathways and process of a successful childhood obesity prevention intervention in Victoria, Australia (25). Brennan et al. (26) and Calancie et al. (27) have employed systems thinking and CBSD with community members and coalitions in U.S. cities to identify strategies for childhood obesity prevention in communities. An additional finding from community coalition work suggests that increases in systems thinking among coalition members fostered unprompted understanding of and attention to health equity concepts and potential related actions (28). As is illustrated across these studies, and expanded upon with the current study, systems approaches reinforce the importance of having a combination of mutually reinforcing interventions, which aligns with current evidence that implementing one intervention, especially in isolation, is not enough (15, 49–51). Furthermore, in the context of multi-factorial or multi-level interventions, understanding the potential pitfalls and unanticipated leverage points within a complex system from the beginning can potentially lead to greater equity impact. For example, findings of this analysis could apply to equity-focused implementation of extant universal feeding programs, which are now recommended by Centers for Disease Control Community Guide (52). Our systems analysis also emphasizes the complexity of effectively implementing these types of school-based interventions.

### 4.1 Strengths and limitations

One strength of this retrospective analysis is that it focuses on a PSE approach, universal school meals, which has an intrinsic focus on equity and has become established practice. The information gleaned from CODP reports and manuscripts provides details on four focal initiatives, their engagement with the context and priority populations, and the ways in which the programs evolved over time within the schools and communities. Additionally, the CODP documentation included data collection during both the pre-study and active study periods, which helped with the development of the systems map.

This analysis also has notable limitations. Given that the case study was an associational study, the data collection and declines in obesity prevalence were concurrent but were not able to be linked to certain schools, individual children or students' food consumption. Also, there might have been other initiatives or external factors occurring concurrently in the community outside of the set boundaries that may have potentially impacted the outcomes and ultimately, the assumptions made in the systems mapping component (e.g., other benefits impacting a family's economic status such as child tax credits). Lastly, there were no CODP data available to analyze students' dietary intake or that addressed contextual factors not directly related to food or obesity that might have affected a student's need or willingness to receive free meals.

## 5 Conclusion

This analysis supports the use of systems mapping tools to augment application of a specific, obesity-focused health equity framework. This analytic approach added value to the original CODP case study (29) because it clearly illustrated the complex interactions that were integral to making the equity portion of the Universal Feeding Pilot intervention work and validated the reasoning of Philadelphia public health leaders and other key informants that the coordinated strategies made sense for working toward equity in the long run. Beyond specific learnings from this particular retrospective analysis, we see several implications for the broader field of PSE change efforts in obesity prevention. Perhaps foremost is the need for broader documentation to include factors less closely linked to the interventions or initiatives of interest but important to the effectiveness of and ability to sustain the relevant programs or policies (i.e., those called for in GTE quadrants 3 and 4). Potentially relevant information might relate to assets or liabilities in the socio-cultural, public policy, economic or for-profit domains. Such information could be identified and documented during partner-engaged needs assessment and planning for new PSE interventions.

Another clear implication is the need to give more priority to implementing systems science approaches in the PSE sphere—to move from talk to action. This might include incentivizing (with funding priority and resources) the use of systems science applications in health-equity research and identifying other health equity frameworks as models amenable to being enhanced by systems approaches. Training programs to enable research capacity in systems applications exist but could be made more accessible and promoted. Additionally, systems science approaches could enable combinations of PSE and individual behavior change approaches, given that changes in population behaviors are ultimate targets of PSE approaches. PSE and lifestyle change approaches both clearly require integrated approaches, especially from an equity perspective, in which disparate exposures and experiences affect both settings and people.

## Data Availability

The original contributions presented in the study are included in the article/Supplementary material, further inquiries can be directed to the corresponding author.

## Footnotes

^1^The term “priority populations” as defined by the National Institutes of Health, National Institute of Minority Health and Health disparities is used in this article to refer to populations given priority for equity-focused research strategies including racial and ethnic minority groups, people with lower socioeconomic status (SES), underserved rural communities, sexual and gender minority (SGM) groups, and people with disabilities (3).

^2^In this article terminology for racial and ethnic categories aligns with how the original reporting source classified these data and thus varies in our usage. We recognize that terminology for racial and ethnic identity is fluid and evolving (12) and so may differ from the categorizations represented here.

^3^NCCOR was formed in 2009 as a collaboration among major U.S. health and nutrition research funders: the Centers for Disease Control and Prevention (CDC), the National Institutes of Health (NIH), the Robert Wood Johnson Foundation (RWJF), and the U.S. Department of Agriculture (USDA). NCCOR's ongoing objective is to accelerate progress in reducing childhood obesity in America.

^4^A system is defined as a set of interrelated elements that interact to achieve an inherent or ascribed purpose (41).
