# Supplementary material for: Using system dynamics mapping to explore synergy in an equity-focused obesity prevention framework
Source: Front Public Health. 2025 Feb 20;13:1525224. doi: 10.3389/fpubh.2025.1525224 (PMC11884243; doi:10.3389/fpubh.2025.1525224)
Supplement: Supplementary file 1 [file Table_1.docx]

Supplementary Material

# Supplementary Tables

**Supplementary Table 1. Descriptions of the four strategies used for the systems mapping analysis**

| **UNIVERSAL SCHOOL FEEDING PILOT (UFP)** | |
| --- | --- |
| **Key Features** | **Facilitators and Partner Roles (+) and Barriers(-)** |
| - Launched by SDP in 1991; first USDA-authorized school pilot. - School-wide eligibility based on SDP estimated high percentage of students would meet income or other eligibility criteria for free- or reduced-price meal - Reduced administrative burden for schools and parents/guardians. - Streamlined the process of counting and claiming meals served to low-income students via the National School Lunch and School Breakfast Programs. - Enabled many children to receive free and reduced-cost meals. - Assuring food security for low-income children was more of a primary focus for the strategy but increasing number of students participating meant that later improvements in food quality reached more low-income students, including those at higher risk for obesity. | - Met multiple goals for the SDP: decreased administrative burden; decreased stigma associated with school meals; increased participation increased food access; per-meal reimbursements to the SDP could be applied to cover operating costs; encouraged wider adoption of the UFP approach. - Mobilized broad civic and political support for preserving the program in light of a threat of discontinuation by the USDA and criticism that the universal feeding approach was an inappropriate use of federal dollars. - Partners had a key role in assuring children’s access to meals by holding the SDP accountable operationally, e.g., implementation of reimbursements to the SDP.   ….…………………   - Attitudes of some principals that parents – rather than schools – were responsible for providing breakfast. - Emphasis on students having breakfast at school was seen as having pros and cons; cons were concerns in relation to competition with other valued aspects of children’s schedules. - Lack of full-service kitchens in 70% of schools decreased meal quality and appeal of the meals. - Unappealing meals would result in plate waste and students resorting to less healthy options such as vending machines or at corner stores. - Lag in awareness that tray count was not equivalent to food consumption. |
| **EAT RIGHT NOW (ERN) Nutrition Education Program** |  |
| **Key Features** | **Facilitators, Partner Roles (+) and Barriers (-)** |
| - Statewide program; part of the Pennsylvania Nutrition Education TRACKS. - Initiated by SDP in 1999 to provide K-12 nutrition education in all schools with at least 50% of students eligible for free-or reduced prices meals. - Funded by the Supplemental Nutrition Assistance Program (SNAP Ed). - SDP, primarily, and others also contributed support; SDP support was mostly in kind—salaries for teachers and nurses who delivered the program. - All eligible schools participated; child participation required for offering schools. - Reached ~ 90% of public-school children annually; overall ~180,000 children received education during the study period. - Also provided community-level education, sometimes paired with selling fruits and vegetables in community setting partnering with the local food bank. | - Educators were committed, creative and experienced, especially in early childhood setting. - Educational programs are enjoyable and well-received by children. - Various academically-based or non-profit partners helped to deliver the program in a set of assigned schools. - Partners had to follow a curriculum that aligned with certain guidelines but had flexibility in educational approaches.   ……………………   - Managing the network of partners was complex and required the PA TRACKS program at Penn State University to manage this aspect. |
| **BAN ON SUGARY DRINKS** | |
| **Key Features** | **Facilitators, Partner Roles (+) and Barriers (-)** |
| - A proposed $43 million exclusive soft drink “pouring contract” for schools to serve only certain brands mobilized community countermeasures. - In 2001, The Food Trust responded by forming a Comprehensive School Nutrition Policy Task Force with over 40 groups and individuals, including the SDP. - In 2004, Philadelphia removed all SSB from vending machines and school cafeterias (a first in the U.S.). - The School Board concurrently introduced snack guidelines to be implemented by 2006. - Also in 2004, the Congressional reauthorization of the Child Nutrition and Special Supplemental Nutrition Program for Women, Infants, and Children (WIC) included a requirement that schools participating in the National School Lunch and other child nutrition programs create local school wellness policies by 2006. | - Implementation of the sugary drink ban emerged through collaborative discussions among cross-sector partners, of whom many were already engaged with and had credibility within the school system. - The Food Trust received a federal grant to expand and evaluate the effectiveness of nutrition and physical activities policies being piloted in some schools, in partnership with Temple University researchers over the two years just prior to the CODP study period. Children in intervention schools were half as likely to become overweight in the two-year period. - The pilot study supported applying the policies to the subsequent Comprehensive District Wide Wellness policies effected in 2006 (see below). - Partners were strong advocates for compliance with the school wellness policies   ……………………   - Schools were hesitant about removing popular (but often unhealthy) vending machine items. |
| **COMPREHENSIVE, DISTRICTWIDE SCHOOL WELLNESS POLICY** | |
| **Key Features** | **Facilitators, Partner Roles (+) and Barriers (-)** |
| - Mandatory policy went into effect in 2006 and was heavily based on the piloted projects. - Established Coordinated School Wellness Councils; nutrition standards for all foods in the school environment during the day; nutrition education and physical activity curricula; physical activity; regular “movement breaks” at intervals during the day for elementary students. - Other activities designed to create and maintain a non-stigmatizing atmosphere related to health and wellness. | - Cross-sector partners involved in School Nutrition Task Force activities included The Food Trust, Temple University, partners within the school sphere as well as city officials and policy makers, and connections to other community organizations promoting healthy change. - The Task Force was critical to the development of the pilot project in collaboration with the SDP, social marketing activities to raise broad awareness of the Wellness Policy and ensuring that all school foods met nutrition standards. - SDP leadership was very supportive of the wellness policies and the trade-off of foregoing profits from the pouring contract in favor of promoting policies to improve children’s health. - Partners were critical for eventually fostering acceptance of and compliance with the requirement for serving and selling healthier items. - CDC guidelines were available to support policy recommendations.   ……………………   - Initial resistance of food service administrators to change and to the Wellness Policy guidelines |
| Source: Dawkins-Lyn N, Greenberg M. Signs of progress in childhood obesity declines. National Collaborative for Childhood Obesity Research (NCCOR). Available: <https://www.nccor.org/downloads/CODP_Site%20Summary%20Report_Philadelphia_public_clean1.pdf> (Accessed on 02-03-2025) | |

**Supplementary Table 2**. Individual Loop Descriptions within the Systems Map

| Loop Name | Loop Description | Visualization |
| --- | --- | --- |
| Loop 1: B1: Intervening on Obesity | This case study reports on the Childhood Obesity Declines project, where the target was the child obesity rate in Philadelphia. Because there are disparities in the prevalence of childhood obesity, where Black children have higher obesity rates, the Universal Feeding Pilot (UFP) was implemented in Philadelphia public schools to increase the consumption of healthy food which would ultimately contribute to reducing the child obesity rate in Philadelphia and the child obesity gap in Philadelphia. | 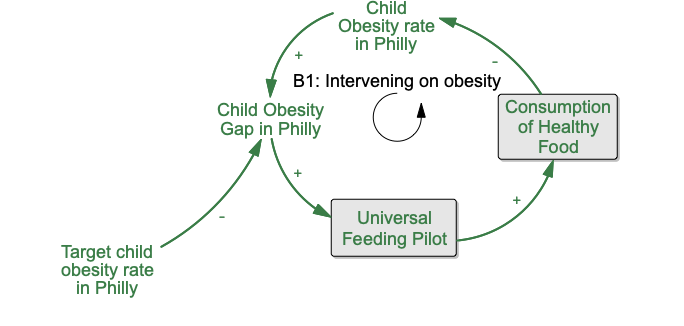 |
| Loop 2: R1: Destigmatizing School Meals | The UFP was universally offered to students in participating schools, thus reducing the stigma for students participating in this program. This in turn increased access to the school meals and to healthier food options for those enrolled in the UFP. | ­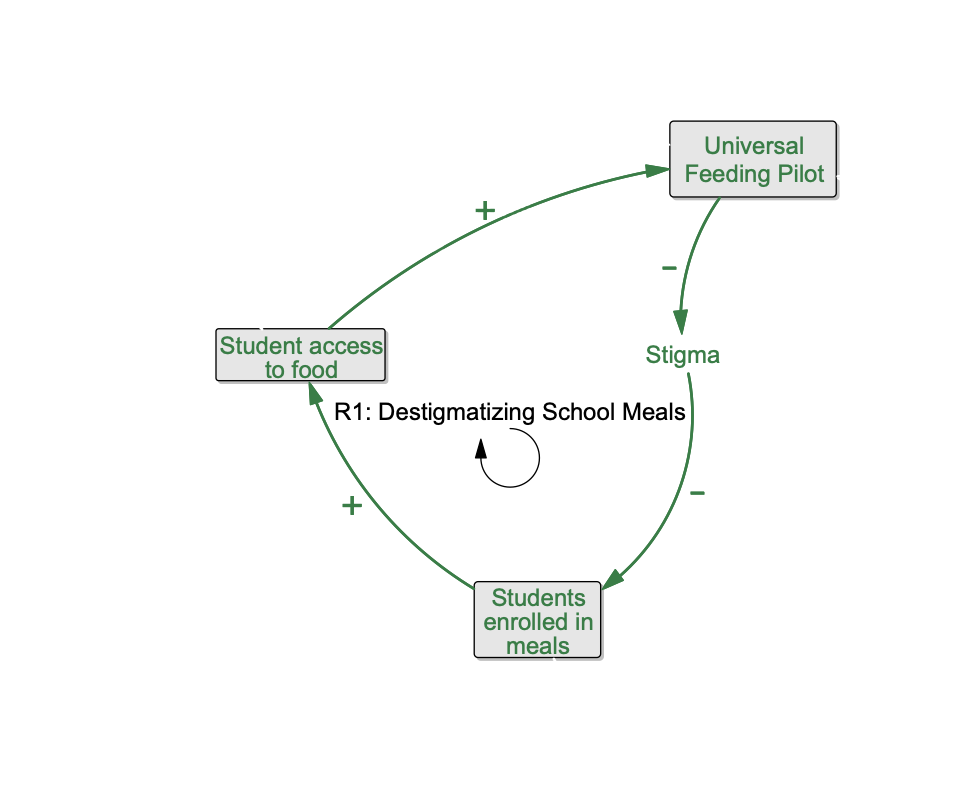 |
| Loop 3: R2—Improving Enrollment | One factor that contributed to enrollment in the UFP was related to the amount of time that families spent on completing the enrollment process. This loop indicates that the paperwork and enrollment processes for the UFP reduced the time that families’ spent completing the enrollment process, which impacted the number of students who were enrolled in the program, and thus had increased access to healthier foods through the UFP. | 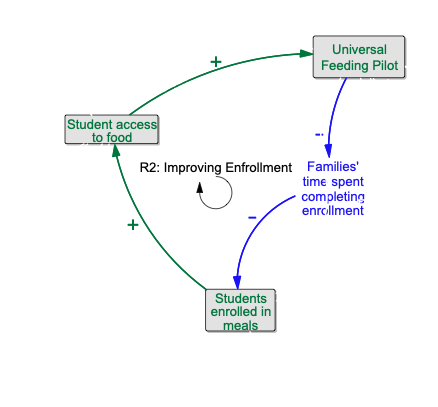 |
| Loop 4: R3: Improving Efficiency | One of the other factors associated with the UFP in public schools is the ways in which the streamlined implementation process of the pilot alleviated some of the administrative burden in participating schools. Therefore, over time, more schools were able to participate in the pilot because there was less capacity strain on the staff of participating schools. | 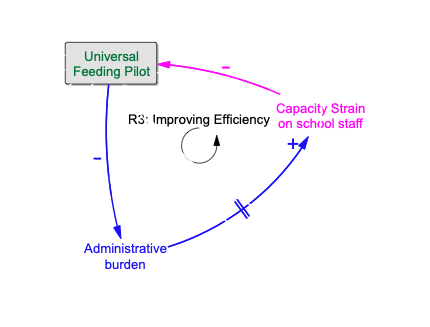 |
| Loop 5: R4—Reducing Administrative Burdens | Another factor that affected enrollment, is that the UFP reduced the administrative burden that the school staff experienced, which mitigated any barriers for students enrolling in the meal program, ultimately increasing their access to the healthier meal options that the pilot offered. | 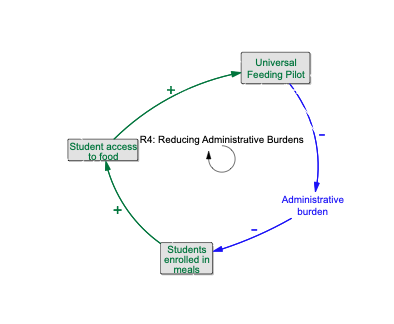 |
| Loop 6: B2-4: Building Food Access | UFP, implemented to address the gap in the observed versus desired child obesity rate, operates through three pathways of reducing stigma, administrative burden, and families’ time spent completing enrollment, to increase the students enrolled in meals. Students enrolled in meals (similar to the pathway in Loop 5: R4) in turn increases student access to food, decreasing food insecurity, which increases the consumption of healthy food. This feeds back to decrease the child obesity rate and decrease the gap driving the need for the UFP in the first place. | 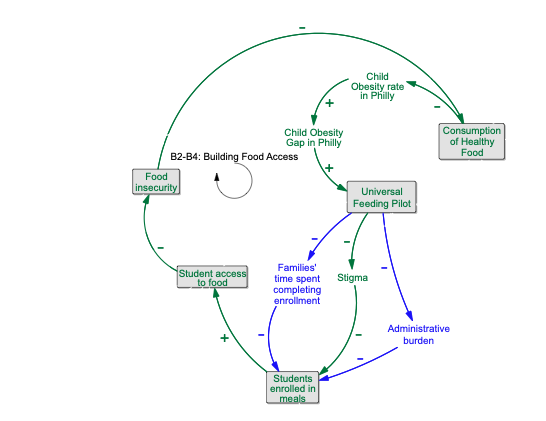 |
| Loop 7: R5—Financial Impact on Capacity | The administrative burden of the implementation of the UFP also impacted the potential financial benefits for the SDP, which ultimately helped relieve some of the capacity strain on the school staff but also provided opportunities for the SDP to save money that could be reallocated for other purposes, such as operating costs. | 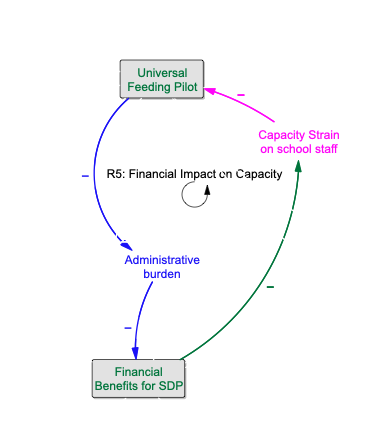 |
| Loop 8: R6—Financial Impact on Administration | In looking at the smaller loop to identify the financial impact on the administration of schools participating in the UFP, we see a reinforcing relationship between the financial benefits for the SDP and the administration burden. This loop depicts how the financial benefits mitigated some of the administrative burden, which was then reduced because of the provision of financial benefits for SDP because of the reduce administrative burden that allowed for the SDP to save money that could be used towards overhead costs. | 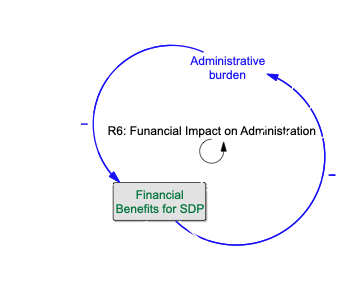 |
| Loop 9: R7&8—Alleviating Capacity Strain | Building on some of the previous smaller loops, this loop shows the mechanism by which capacity strain was alleviated through the implementation of the UFP. By streamlining the process for identifying student eligibility, reviewing and processing student paperwork, and destigmatizing participation in the program, more students were enrolled in the pilot. This led to an increase in the financial benefits for SDP, which ultimately contributed to relieving some of the capacity strain that the staff experienced at participating schools. | 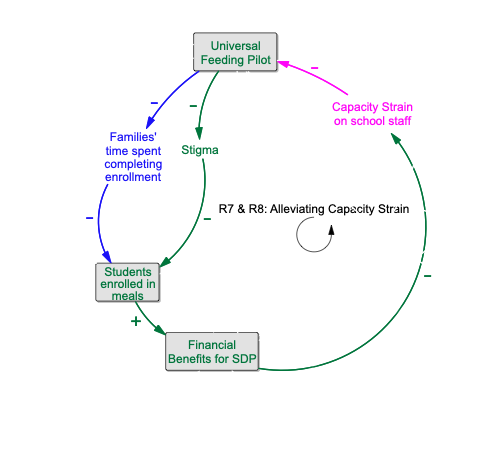 |
| Loop 10: R9-12—Accumulating Support | Another factor that the financial benefit for SDP contributes to is the collaboration of partners on shared interests in the program and its goals. This collaboration increased the willingness for organizations to partner together, leading to the development and mobilization of the Childhood obesity task force. This task force supported the UFP but also branched out to include advocacy efforts organizing against sugar sweetened beverage policies and access in Philadelphia schools, especially those that participated in the UFP program. | 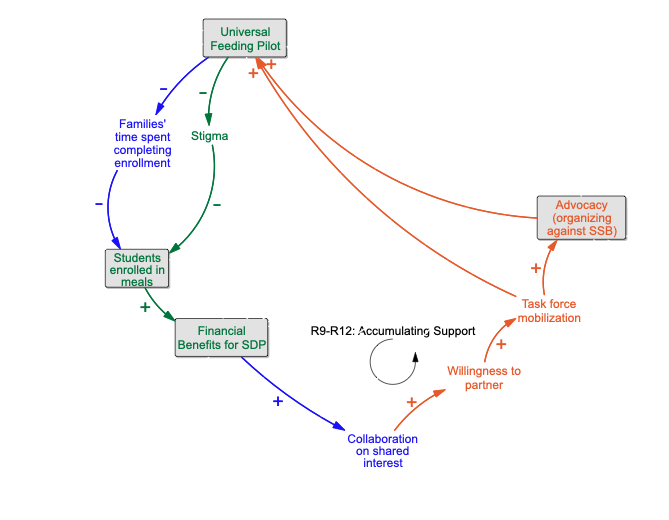 |
| Loop 11: B5-8—Burden of Delivery Costs | This loop incorporates another factor that impacted implementation and students’ access to food, and that’s the reliance on delivery of the meals through a third-party vendor. The delivery of the foods was suppose to increase student access to healthier meal options, however this reliance cut into some of the financial benefits for SDP which ultimately impacted the administrative burden and the capacity strain on the school staff. This unintended consequence contributed to an accumulation of administrative burden, so while the effects were not immediate on capacity strain, over time it did impact the school staff at the schools participating in the UFP. | ­­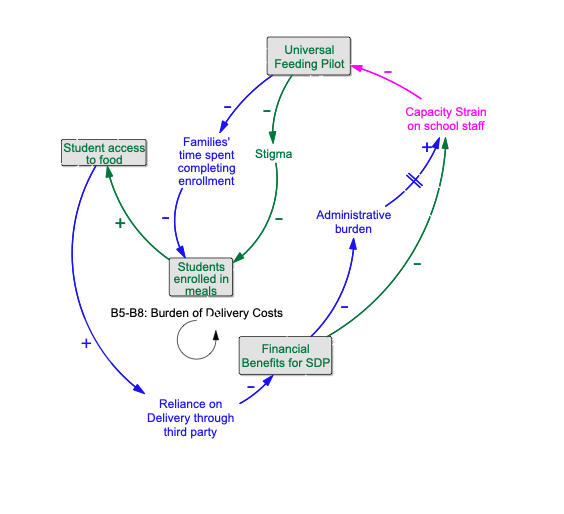 |
| Loop 12: B9-14—Mismatch in Food Preferences | Building on Loop 11: B5-8, other unplanned consequences of implementing the UFP program were that students’ participation in the program and access to the food options led to a mismatch in food preferences, leading to an increase in plate waste and an increase in opinions on the healthier options being unappealing. Both factors ultimately led to students resorting to alternative food sources to supplement the meals (or rather, what they did not eat), ultimately leading to consumption of unhealthy foods in the school cafeterias or vending machines. This unhealthy food consumption decreased the financial benefits for SDP since children were spending money elsewhere and more money had to be spent towards making the meals more appealing to participating students, and a reduction in financial benefits negatively impacted the capacity strain on school staff. | 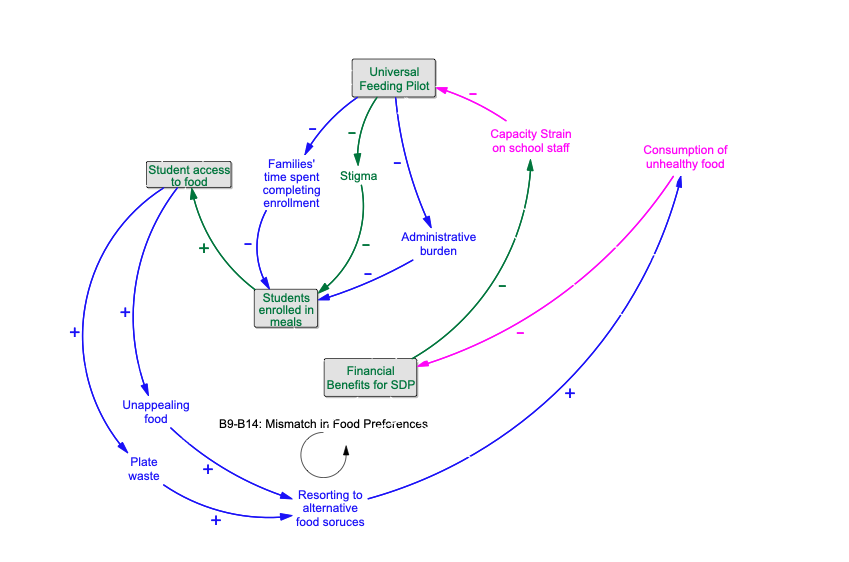 |
| Loop 13: B15-18—Alleviating Burden for Families | One of the goals of the UFP was to alleviate the financial burden for Philadelphian families in procuring healthier food options for their school-aged children. Increasing students’ access to healthy foods would reduce the need for the families to feed the students at home, reducing the amount of money that families would spend on healthy food options which would improve the financial burden and reduce food insecurity. This access would ultimately reduce food insecurity by the increased consumption of healthy food and lead to a reduction in the child obesity prevalence in Philadelphia. | 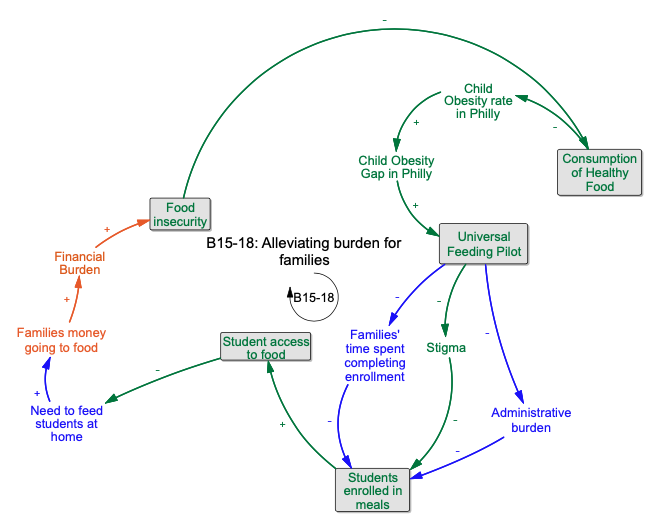 |
| Loop 14: R13-18—Alleviating Families’ Time | The need for families to feed their children at home also reduced the ability to reallocate time to other needs and activities. When families no longer had to do this, they potentially could allot that time to more multi-sector support, increasing the collaboration on shared interest and the willingness for organizations to establish partnerships and subsequently partners’ participation in the task force. | ­­­­­­  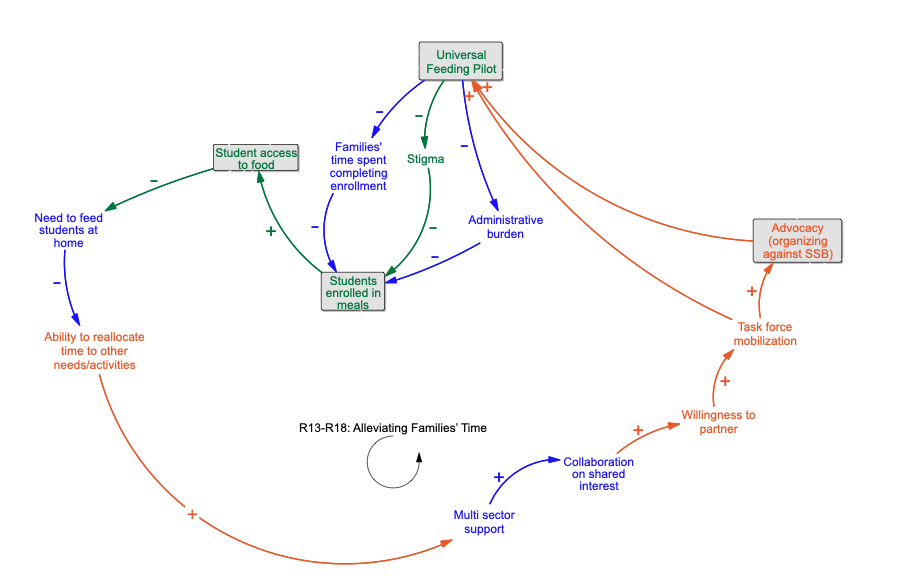 |
| Loop 15: R19-21—Consolidating Support | Building on Loop 10: R9-12, mobilization of the task force and the task force’s advocacy efforts organizing against sugar sweetened beverage availability and access in schools also increased the ability to build multi-sector support and allowed for the identification and collaboration with other partners who were interested in contributing to the cause. | 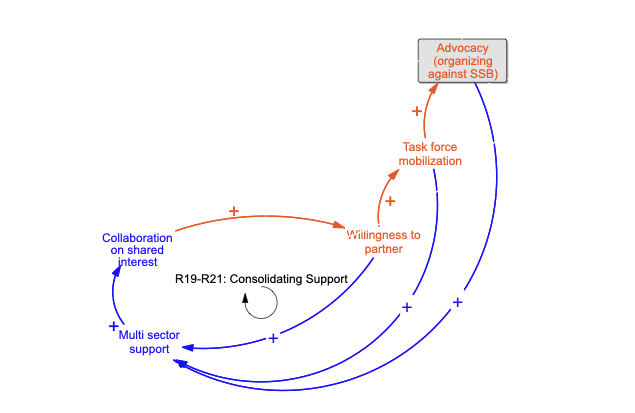 |
| Loop 16: B19-24—Shifting the Nutrition Context | Collaboration with program partners with shared interest created a shift in the nutrition context of Philadelphia public schools and led the development of a Comprehensive District Wellness Plan, which implemented a set of nutrition standards to increase the consumption of healthy foods in schools while simultaneously decreasing consumption of unhealthy foods. These school nutrition standards and policies ultimately contributed to reducing the child obesity prevalence among school-aged children in Philadelphia. | 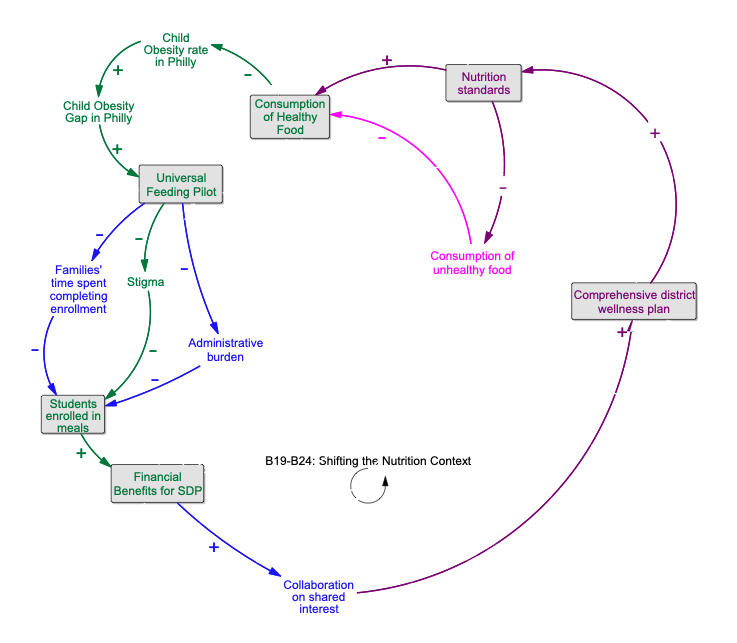 |
| Loop 17: R22&23—Changing Norms around Obesity | Building on Loop 16: B19-24, the nutrition standards that were implemented in participating schools as part of the Comprehensive District Wellness Plan led to a delayed effect that changed the norms and resources for food access in Philadelphia schools. This loop also demonstrates that the child obesity gap also had a delayed effect on the norms around food access and obesity allowing for changes in norms and resources for food would allow for schools to continue to implement and evolve the comprehensive district wellness over time as the obesity trends changed. | 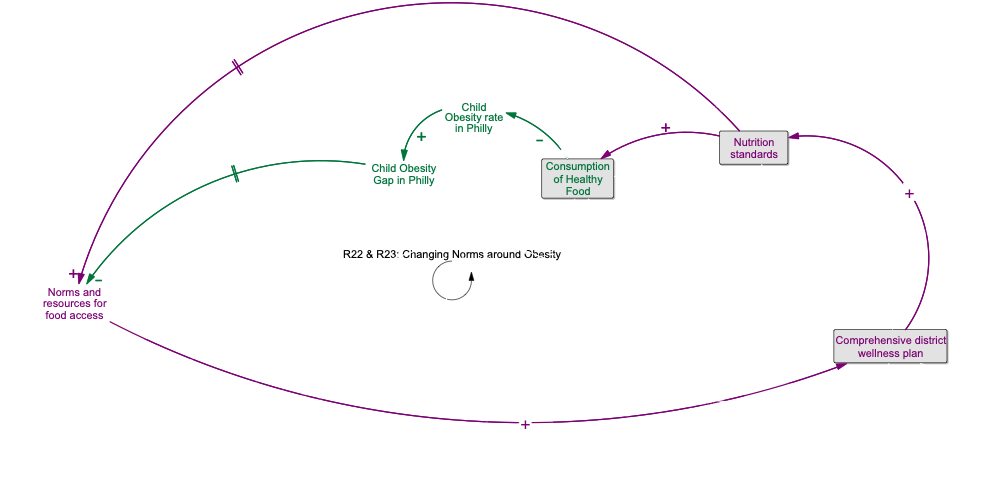 |

**
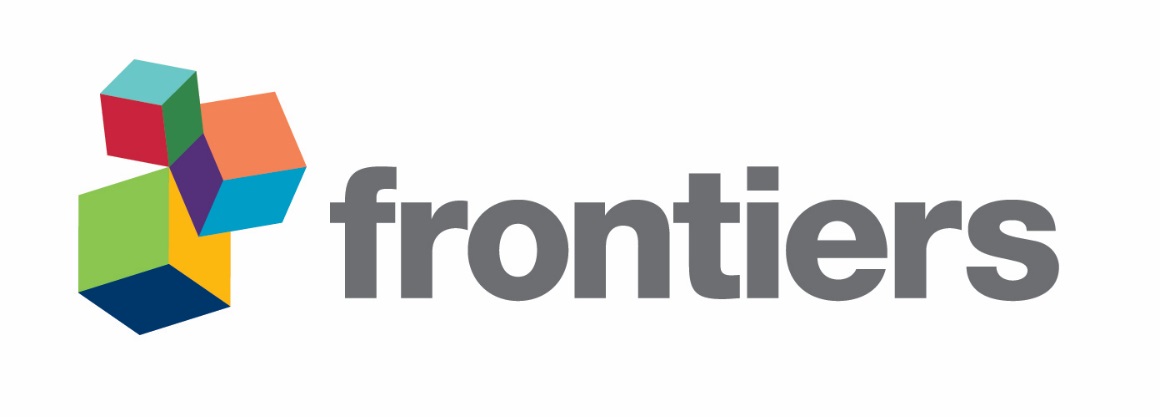
**
